# Shape-Shifted Red Blood Cells: A Novel Red Blood Cell Stage?

**DOI:** 10.3390/cells7040031

**Published:** 2018-04-19

**Authors:** Verónica Chico, Sara Puente-Marin, Iván Nombela, Sergio Ciordia, María Carmen Mena, Begoña Carracedo, Alberto Villena, Luis Mercado, Julio Coll, María del Mar Ortega-Villaizan

**Affiliations:** 1 Instituto de Biología Molecular y Celular, Universidad Miguel Hernández, 03202 Elche, Spain; vchico@umh.es (V.C.); spuente@umh.es (S.P.-M.); inombela@umh.es (I.N.); 2 Unidad de Proteómica, Centro Nacional de Biotecnología (CNB-CSIC), 28049 Madrid, Spain; sciordia@cnb.csic.es (S.C.); mcmena@cnb.csic.es (M.C.M.); 3 Área de Biología Celular, Departamento de Biología Molecular, Universidad de León, 24071 León, Spain; bcarr@unileon.es (B.C.); alberto.villena@unileon.es (A.V.); 4 Instituto de Biología, Pontificia Universidad Católica de Valparaíso, 2373223 Valparaíso, Chile; luis.mercado@pucv.cl; 5 INIA-SIGT–Biotecnología, 28040 Madrid, Spain; juliocoll@inia.es

**Keywords:** rainbow trout, red blood cells, shape-shifted red blood cells, RNA-seq, transcriptome, proteome, functional network, immune response, heat-stress, VHSV

## Abstract

Primitive nucleated erythroid cells in the bloodstream have long been suggested to be more similar to nucleated red cells of fish, amphibians, and birds than the red cells of fetal and adult mammals. Rainbow trout Ficoll-purified red blood cells (RBCs) cultured in vitro undergo morphological changes, especially when exposed to stress, and enter a new cell stage that we have coined shape-shifted RBCs (shRBCs). We have characterized these shRBCs using transmission electron microscopy (TEM) micrographs, Wright–Giemsa staining, cell marker immunostaining, and transcriptomic and proteomic evaluation. shRBCs showed reduced density of the cytoplasm, hemoglobin loss, decondensed chromatin in the nucleus, and striking expression of the B lymphocyte molecular marker IgM. In addition, shRBCs shared some features of mammalian primitive pyrenocytes (extruded nucleus surrounded by a thin rim of cytoplasm and phosphatidylserine (PS) exposure on cell surface). These shRBCs were transiently observed in heat-stressed rainbow trout bloodstream for three days. Functional network analysis of combined transcriptomic and proteomic studies resulted in the identification of proteins involved in pathways related to the regulation of cell morphogenesis involved in differentiation, cellular response to stress, and immune system process. In addition, shRBCs increased interleukin 8 (IL8), interleukin 1 β (IL1β), interferon ɣ (IFNɣ), and natural killer enhancing factor (NKEF) protein production in response to viral hemorrhagic septicemia virus (VHSV). In conclusion, shRBCs may represent a novel cell stage that participates in roles related to immune response mediation, homeostasis, and the differentiation and development of blood cells.

## 1. Introduction

It has recently come to light that nucleated red blood cells (RBCs) of fish are multifunctional cells, because in addition to being involved in gas exchange and transport, it also has been reported that they respond to pathogens. Rainbow trout RBCs take part in the host defenses against fungal infections [1] and also have membrane receptors for pathogen associated molecular patterns (PAMPs) and produce cytokine transcripts [2,3]. In addition, piscine RBCs produce immune responses when infected by replicating viruses, such as infectious salmon anemia virus (ISAV) [4] or piscine orthoreovirus (PRV) [5], or by nonreplicating viruses like viral hemorrhagic septicemia virus (VHSV) [6] and infectious pancreatic necrosis virus (IPNV) [7,8]. Recently, it was reported that turbot RBCs produce Nk-lysin (Nkl), an antimicrobial peptide involved in the resistance against VHSV [9]. In addition, rainbow trout RBCs can engulf cells of the ubiquitous fungal species *Candida albicans* and present them to macrophages [1]. Moreover, rainbow trout RBCs have been described to exert paracrine molecular antiviral communication with other cells [6]. This evidence indicates that fish RBCs importantly contribute to immune response to infections [8]. Similarly, human cord blood nucleated RBCs have been shown to exert a regulatory function in the innate immune response, by means of the suppression of the production of inflammatory cytokines such as tumor necrosis factor α (TNFα) and interleukin 1 β (IL1β) from monocytes in response to lipopolysaccharide (LPS) [10]. 

Other roles such as modulation of inflammation, angiogenesis, coagulation and vascular tone have been described for mammalian RBCs (reviewed in Akbari A. 2011) [11]. Separately, transcriptomic analysis of nucleated RBCs of rainbow trout and Atlantic salmon [5,12] revealed the presence of genes related to differentiation and development of blood cells, indicating that nucleated RBCs could be retaining potential for cell differentiation. In mammals, primitive nucleated erythroid cells in circulating blood have long been suggested to be more similar to nucleated red cells of birds, fish, and amphibians than the red cells of fetal and adult mammals [13]. Erythroid cells extrude their nucleus at the end of differentiation, giving rise to a pyrenocyte and a reticulocyte that finally matures to a red cell [14]. Primitive erythroid cells in murine embryo enucleate and continue to circulate for several days after birth [15]; their enucleation leads to a transient population of primitive pyrenocytes in the bloodstream [13]. 

In this report, we describe a novel finding in rainbow trout RBCs. Rainbow trout RBCs cultured in vitro revealed striking morphological changes into what we have termed shape-shifted RBCs (shRBCs). When exposed to certain stimuli, the cells changed their oval shape and nucleus to round, lost their hemoglobin, thinned their membranes, and expressed new molecular markers like IgM. In addition, shRBCs shared some features of mammalian primitive pyrenocytes (extruded nucleus surrounded by a thin rim of cytoplasm, phosphatidylserine (PS) exposure on the cell surface, and engulfment by macrophages [13,16]). In contraposition to mammalian pyrenocytes, which rapidly disintegrate in cell culture [14], shRBCs were highly refractive in in vitro culture for more than a month. In vivo, they appeared in the peripheral blood after heat stress stimulation and remained in the circulation at least 72 h after stimulation. In an attempt to further characterize shRBCs, we performed transcriptomic and proteomic analyses. Functional network analysis of combined transcriptomic and proteomic studies resulted in the identification of proteins involved in pathways such as: (i) regulation of cell morphogenesis involved in differentiation, (ii) cellular response to stress, and (iii) immune system process. On the other hand, shRBCs halted VHSV infection and increased cytokines and the natural killer enhancing factor (NKEF) protein production. Moreover, shRBCs conditioned medium (CM) induced an upregulation of interferon γ (IFNγ)-activated genes and interleukin 8 (*il8*) gene in the TPS-2 (trout pronephros stroma) cell line. 

Taken together, this evidence implies the involvement of a novel RBC stage, shRBCs, in cell differentiation and immune responses. A potential application of this study could be considering shRBCs as mediator cells of the immune response and therefore a potential target for novel DNA vaccines and/or new strategies against fish viral infections.

## 2. Materials and Methods

### 2.1. Animals

Rainbow trout (*Oncorhynchus mykiss*) around 6 to 7 cm in size were obtained from a VHSV-free commercial farm and maintained at the University Miguel Hernandez (UMH) facilities at 14 °C. Prior to experiments, fish were acclimatized to laboratory conditions for 2 weeks. Animal care and all activities involving animal handling and experimentations were done according Spanish [Real Decreto 1201/2005] and EU [EU Directive EC86/609, and Appendix A to Convention ETS123, 2007/526/CE] regulations and recommendations for animal experimentation. All experimental protocols were reviewed and approved by the Animal Welfare Body and the Research Ethics Committee at the UMH and by the competent authority of the Regional Ministry of Presidency and Agriculture, Fisheries, Food and Water supply.

### 2.2. Cell Cultures

Rainbow trout RBCs were obtained and purified as previously described [6]. Briefly, RBCs obtained from the caudal vein were purified by Ficoll density gradient centrifugation (Ficoll 1.007; Sigma-Aldrich, Madrid, Spain). Purified RBCs were cultured in 25 cm^2^ cell culture flasks (Nunc, Roskilde, Denmark) with RPMI-1640 medium (Dutch modification) (Gibco, Thermo Fischer Scientific, Waltham, MA, USA) supplemented with gamma- irradiated 10% FBS (fetal bovine serum) (Cultek, Madrid, Spain), 1 mM pyruvate (Gibco), 2 mM l-glutamine (Gibco), 50 μg/mL gentamicin (Gibco), 2 μg/mL fungizone (Gibco), 100 U/mL penicillin, and 100 μg/mL streptomycin (Sigma-Aldrich) at 14 °C for 24 h before experimentation.

The fish cell line TPS-2 (rainbow trout stromal pronephros cell line) [17], donated by Dr. AJ Villena, was also used in this work. TPS-2 cells were maintained in RPMI medium containing 20% FBS, 1 mM pyruvate, 2 mM L-glutamine, 50 µg/mL gentamicin and 2 µg/mL fungizone at 21 °C.

The EPC (Epithelioma Papulosum Cyprini) cell line [18] was purchased from the Americal Type Culture Collection (ATCC, CRL-2872). EPC cells were maintained in RPMI medium containing 10% FBS, 1 mM pyruvate, 2 mM l-glutamine, 50 μg/mL gentamicin, and 2 μg/mL fungizone at 28 °C.

### 2.3. Transmission Electronic Microscopy (TEM) and Wright-Giemsa Staining

Cells were fixed with glutaraldehyde (Sigma-Aldrich) at 2% in 0.1 M cacodylate (Sigma-Aldrich) buffer for 4 h at room temperature (RT). Post-fixation was done with 1% osmium tetroxide (Electron Microscopy Sciences, Hatfield, PA, USA) in 0.1 M cacodylate buffer for 1 h at 4 °C. Pellets were washed with 0.1 M cacodylate buffer 3 times for 10 min. During the last wash, cells were kept at 4 °C overnight. Samples were added to 3% agar (Sigma-Aldrich). Then, sequential ethanol (Panreac, Barcelona, Spain) washes (30% to 100%) were used to dehydrate the samples, followed by acetone (Panreac), acetone/Epon resin (Electron Microscopy Sciences) 1:1 (1 h) and Epon resin fixation. Ultrathin sections were prepared with a Leica Ultracut ultramicrotome (Leica Microsystems Inc., Richmond Hill, ON, Canada), placed on copper grids (Electron Microscopy Sciences), and stained with uranyl acetate (Electron Microscopy Sciences). Samples were visualized with the Jeol 1011 transmission electron microscope (Jeol Ltd., Tokyo, Japan), and micrographs were taken with Erlangshen ES500W camera (Gatan, Inc., Pleasanton, CA, USA) at the Bioengineering Institute of University Miguel Hernandez.

Slide preparation of Ficoll-purified RBCs incubated at 25 °C for 3 days were fixed with acetone (20 min) and stained with Wright-Giemsa (Sigma-Aldrich) for 10 min. Stained cells were photographed with an inverted microscope (Nikon Eclipse TE2000-U; Nikon Instruments, Inc., Melville, NY, USA) equipped with a digital camera (Nikon DS-1QM).

### 2.4. Flow Cytometry and Immunofluorescence

Cells were fixed, permeabilized, and incubated with primary and secondary antibodies as previously described [6]. Flow cytometry analysis was done by using a FACSCanto II (BD Biosciences, Madrid, Spain) flow cytometer. Immunofluorescence and brightfield images were taken with the cell imaging system IN Cell Analyzer 6000 (GE Healthcare, Little Chalfont, UK). RBC and shRBC populations were selected by forward scatter (FSC) and side scatter (SSC). 

The Annexin V FLUOS staining kit (Roche, Barcelona, Spain) was used for annexin V staining according to manufacturer’s protocol. For nuclei labelling, cells were incubated for 5 min with 4′,6-diamidine-2′-phenylindole dihydrochloride (DAPI) (Sigma-Aldrich) at a concentration of 0.3 pg/mL. 

### 2.5. Antibodies

Rabbit polyclonal antibodies against rainbow trout β-defensin 1 (BD1) [6] and myxovirus resistance protein (Mx) [19,20] were produced at Dr. Amparo Estepa’s laboratory. Mouse polyclonal antibodies agaisnt interleukin 1 β (IL1β) [21], interleukin 8 (IL8) [22], interferon 1(IFN1) [6], interferon γ (IFNɣ) [6] and the natural killer enhancing factor (NKEF) [23] were produced by Dr. Luis Mercado. Myeloid cell surface antigen (CD33) and hematopoietic progenitor cell surface antigen (CD34) antibodies were obtained from CUSABIO Life Science (Houston, TX, USA). Glycophorin C (GYPC) antibody was obtained from Santa Cruz Biotechnology (Madrid, Spain). To label VHSV virions, we used monoclonal murine 2C9 antibody against N protein of VHSV [24]. Monoclonal antibody (1G7) was used to label rainbow trout IgM [25,26]. These antibodies were both donated by Dr. Julio Coll’s laboratory.

### 2.6. Cell Viability Assay

Cell viability was performed using two different methods: Click-iT^®^ RNA imaging kit (ThermoFisher Scientific, Waltham, MA, USA) and LIVE/DEAD^®^ Cell Viability/Cytotoxicity Assay Kit (ThermoFisher).

The Click-iT^®^ RNA imaging assay was done according to the manufacturer’s instructions. Briefly, cells were plated at a density of 10^6^ cells/well and EU (5-ethynyl uridine) solution was added at a 1 mM final concentration for 24 h. Cells were harvested, centrifuged at 1600 rpm for 5 min and fixed with 4% PFA and 0.008% glutaraldehyde for 15 min. Following fixation, cells were permeabilized with saponin (Sigma-Aldrich) buffer for 15 min. Cells were then incubated with 500 μL Click-iT reaction buffer for 30 min and washed with phosphate-buffered saline (PBS). EU is incorporated into nascent RNA; RNA was detected in cells with Alexa Fluor^®^ 488 (green) using the cell imaging system IN Cell Analyzer 6000. 

LIVE/DEAD^®^ Cell Viability/Cytotoxicity Assay Kit was used following the manufacturer’s instructions. Briefly, LIVE/DEAD cell imaging reagents were mixed and added at equal volume (2× stock) to cells, followed by incubation for 15 min at RT. Live cells fluoresce bright green (substrate calcein AM), whereas dead cells with compromised membranes fluoresce red (EthD-1 [ethidium homodimer-1]). Immunofluorescence images were taken with the cell imaging system IN Cell Analyzer 6000.

### 2.7. RNA-seq of RBCs and shRBCs

Ficoll-purified RBCs (2 × 10^6^) from 2 individuals were incubated at 14 °C and 28 °C for 3 days. Then, the 14 °C RBCs and 28 °C shRBCs were sorted by means of single-cell protocol to obtain a homogeneous sample of pure RBCs and shRBCs composed of 20 to 30 cells using the BD FACSJazz flow cytometer sorter (BD Biosciences). Sorted RBCs and shRBCs were visualized using the IN Cell Analyzer 6000. Samples were lysed with 9.5 µL of 10× lysis buffer (Clontech, Takara Bio, Mountain View, CA, USA) and 0.5 µL of RNase Inhibitor (Invitrogen, ThermoFisher Scientific, Waltham, MA, USA), and preserved at −80 °C until cDNA library construction.

RBCs and shRBCs cDNA production, RNA-seq library preparation, sequencing, and mapping were carried out by STABVida Lda. (Caparica, Portugal) as previously described [27].

### 2.8. Proteomic Sequencing

Pure shRBC population from two individuals were obtained after culturing RBCs for a month at 14 °C. 10^6^ cells per fish were pelletized by centrifugation (5 min, 700× *g*). Supernatant was removed, and the cell pellet was digested, cleaned-up/desalted, and subjected to liquid chromatography and mass spectrometry analysis (LC-MS) as previously described [27].

### 2.9. Pathway Enrichment Analysis

ClueGO [28], CluePedia [29] and Cytoscape [30] plugins were used to performed Gene Ontology (GO) and pathway annotation networks of the expressed genes and proteins pathway enrichment analysis. The GO Biological Process pathway database was used. A *p*-value ≤ 0.05 and Kappa score of 0.4 were considered as threshold values. Protein-protein interaction (PPI) networks were analyzed using STRING v10.5 (http://string.embl.de/) [31] with a medium confidence score threshold of 0.4. Genes and proteins were identified by sequence homology with those from Homo sapiens using Blast2GO version 4.1.9 [32]. 

### 2.10. Preparation of CM from shRBC Cell Cultures

Conditioned medium (CM) from RBCs and shRBCs was prepared by incubating 10^7^ Ficoll-purified RBCs in 3 mL of RPMI 10% FBS at 14 °C and 28 °C for 3 days. After incubation, supernatants were collected by centrifugation at 1500 rpm for 5 min and filtereded using 0.2 μm pore size filters (Corning, New York, NY, USA; Sigma-Aldrich). Samples were stored at −20 °C until use.

### 2.11. TPS-2 and shRBCs Infection with VHSV

Viral hemorrhagic septicemia virus (VHSV-07.71) [33] was purchased from the ATCC (VR-1388) and cultured in EPC cells at 14°C as previously described [34].

TPS-2 cells were seeded in 24-well plates and incubated with RBC and shRBC CM, diluted both at 1/5 and 1/125, for 24 h at 21 °C. After that, RNA extraction and RT-qPCR was performed as described [6] using specific primers and probes for IFNγ-activated genes (interleukin 1 β [*il1β*], interleukin 6 [*il6*], interleukin 12 β [*il12β*], interleukin 15 [*il15*], tumor necrosis factor α [*tnfα*], and inducible nitric oxide synthase [*inos]*) and interleukin 8 (*il8*) (Table 1). Separately, CM pre-treated cells were infected with VHSV (multiplicity of infection [MOI] of 10^−2^) in a final volume of 500 μL/well of RPMI 2% FBS at 14 °C for 2 h. Infected cell monolayers were then washed, fresh medium was added, and plates were further incubated for 24 h. VHSV replication in TPS-2 cells was evaluated by RT-qPCR using specific primers and probe sequences for the gene encoding VHSV protein N (Table 1). Non-treated TPS-2 cells infected with VHSV were included as a control.

The shRBCs obtained after incubation of RBCs at 28 °C for 3 days were exposed to VHSV (MOI 100) at 14 °C. After 3 h of incubation with VHSV, shRBCs were washed with cold RPMI, then RPMI 2% FBS was added and the culture was incubated at 14 °C for 3 days. Finally, shRBCs were subjected to immunostaining and flow cytometry. The shRBC population was selected by FSC and SSC.

The supernatant of shRBCs exposed to VHSV was analyzed by a focus forming units assay in EPC cells to determine VHSV titer. Serial dilutions of supernatant (10^−1^ to 10^−4^) were added to EPC cells for 2 h. Then, cell monolayers were washed with RPMI 2% FBS and incubated at 14 °C for 24 h. After that, VHSV foci were determined by using an immunostaining focus assay as previously described [7]. N-VHSV antibody was used as primary antibody. Immunofluorescence images were taken using the IN Cell Analyzer 6000. 

### 2.12. Rainbow Trout In Vivo Heat Stress

Rainbow trout of 9 to 11 cm were subjected to heat stress as described in Rendell et al. [40]. Individuals were exposed to 25 °C for 1 h. After that, they were returned to normal temperature (14°C) and were bled at 1, 24, 48 and 72 h. Blood samples (200µL) were diluted 1/10 in RPMI 10% FBS with 100 U/mL penicillin and 100 μg/mL streptomycin and cultured in 96-well plates at a density of 10^5^ cells/well. Cells were incubated for 30 min at 14 °C to allow them to settle to the bottom of the wells.

For DAPI staining, blood cells were incubated for 10 min with 0.3 pg/mL DAPI to label the nuclei of shRBCs. Images were taken using the IN Cell Analyzer 6000. DAPI-positive cells were counted using the IN Cell Analyzer 6000 workstation 3.7.2 software (GE Healthcare, Little Chalfont, UK).

### 2.13. RBCs Exposed to Different Pathogen-Associated Molecular Patterns (PAMPs)

Ficoll-purified RBCs were exposed for 3 days at 14 °C to different PAMPs: (i) VHSV at MOI 1, (ii) laminarin (Sigma-Aldrich) (0.5 µg/µL), (iii) Lipopolysaccharide (LPS, Sigma-Aldrich) (1 µg/µL), and (iv) polyinosinic-polycytidylic acid (Poly I:C, Sigma-Aldrich) (30 µg/µL). Cells were stained with 0.3 pg/mL DAPI for 10 min. Immunofluorescence images were taken with the IN Cell Analyzer 6000. DAPI-positive shRBCs were counted using the IN Cell Analyzer worstation 3.7.2 software.

### 2.14. Software and Statistics

Graphic representation and statistics calculations were performed by Graphpad Prism 6 (www.graphpad.com). The statistical test used and the *p*-values obtained are indicated in the results for each assay. Flowing Software (www.flowingsoftware.com/) was used to analyze flow cytometry data.

## 3. Results

### 3.1. Rainbow Trout RBC Transformation Sequence to shRBC In Vitro

**Figure 1:**
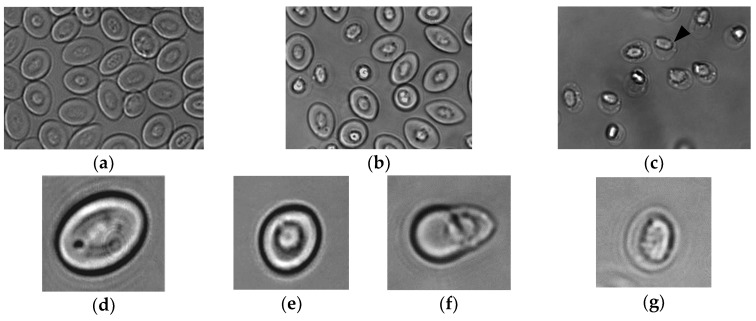
Rainbow trout RBC transformation sequence to shRBC induced by temperature increases. Ficoll-purified RBCs were incubated for 3 days at different temperatures: (**a**) 14 °C, (**b**) 25 °C and (**c**) 28 °C. The black arrow indicate shRBCs. Representative bright-field microscopy images of the sequence of morphological changes in RBCs were taken with 60× magnification: (**d**) RBC, (**e**) stressed RBC, (**f**) round RBC with extruding nucleus and (**g**) shRBC.

The sequence of morphological changes in rainbow trout Ficoll-purified RBCs obtained from the peripheral blood were observed in in vitro cultures by bright-field microscopy. After 3 days of culture at 25 °C some of the initially oval RBCs (Figure 1a) became gradually rounded, smaller, and highly refractive—changes that ultimately resulted in a small, round cell with a thin and almost transparent membrane. We called these cells shape-shifted RBC (shRBC) (Figure 1c). Moreover, in vitro shRBC cultures maintained their characteristic refractive aspect for more than a month. However, the morphological evolution of RBCs varied among individuals. In order to determine a physiological condition that triggered the morphological transformation process, Ficoll-purified RBCs were incubated at different temperatures (14 °C, 25 °C and 28 °C), because we had previously observed that high temperatures alter rainbow trout RBC morphology. Figure 1a–g shows the most typical changes over time from RBC to shRBC morphology. Morphological changes at 25 °C and 28 °C were similar, but transformation was faster and more abundant as the temperature increased. We observed high numbers of RBCs at 14 °C, a mixed population of RBCs and transformed shRBCs at 25 °C, and high numbers of shRBCs at 28 °C. On the other hand, RBCs exposed to different physiological stresses such as PAMPs exposure exhibited low but detectable numbers of shRBCs (Supplementary Figure S1). These results confirmed that when exposed to certain stressors, in vitro cultured RBCs shift to a different cell stage: shRBCs.

### 3.2. Morphological Characterization of Rainbow Trout shRBCs

**Figure 2:**
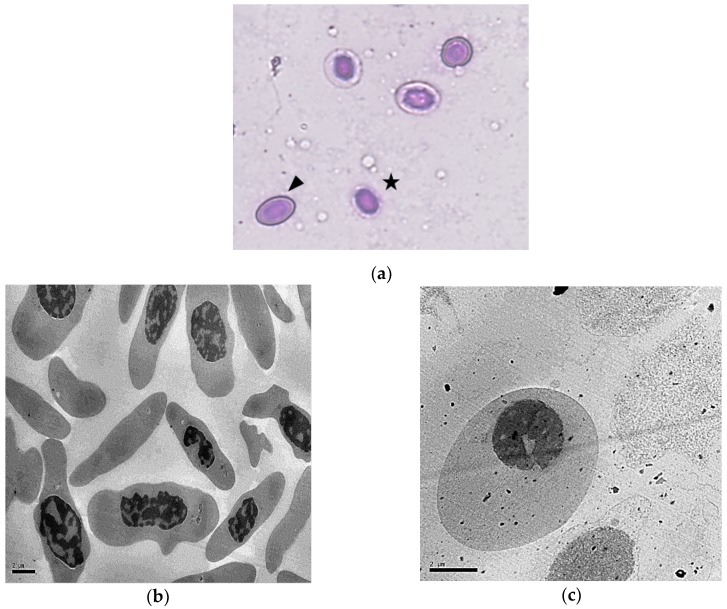

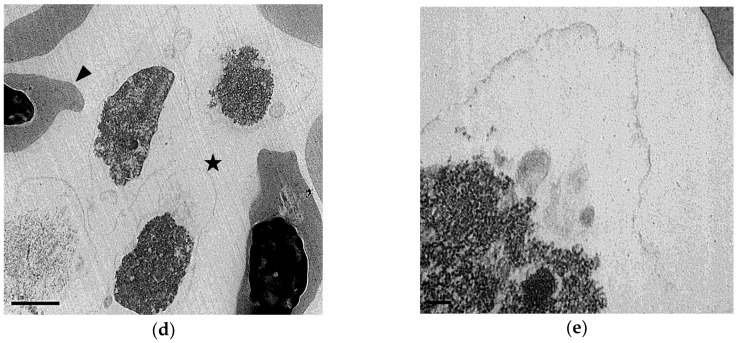
Characterization of rainbow trout RBC transformation to shRBC. Ficoll-purified RBCs were incubated at 25 °C for 3 days. (**a**) Representative images of Wright–Giemsa staining were taken with 40× magnification and are shown, along with TEM micrographs of (**b**) characteristic RBCs, (**c**) stressed RBCs and (**d**, **e**) shRBCs. Black arrowhead: RBC; black star: shRBC. TEM images were taken with: (**b**) 5k×, (**c**) 8k×, (**d**) 10k×, and (**e**) 50k× magnifications.

Wright–Giemsa staining of shRBCs resulted in light cytoplasmic staining and a dark-blue stained, round, expanded nucleus (Figure 2a, black star). In contrast, Wright–Giemsa staining of RBCs resulted in a dark pink cytoplasm and an oval, light blue condensed nucleus (Figure 2a, black arrow).

Characterization of shRBCs by TEM and Wright–Giemsa staining was performed. TEM micrographs of shRBCs confirmed the presence of a thinner membrane surrounding a rounded nucleus with cytoplasm of reduced density (Figure 2d), compared to RBCs (Figure 2b) or stressed RBCs (Figure 2c). The lower-density shRBC cytoplasm is indicative of hemoglobin loss. In addition, shRBCs showed some phagosome-like vesicles around the nucleus (Figure 2e).

**Figure 3:**
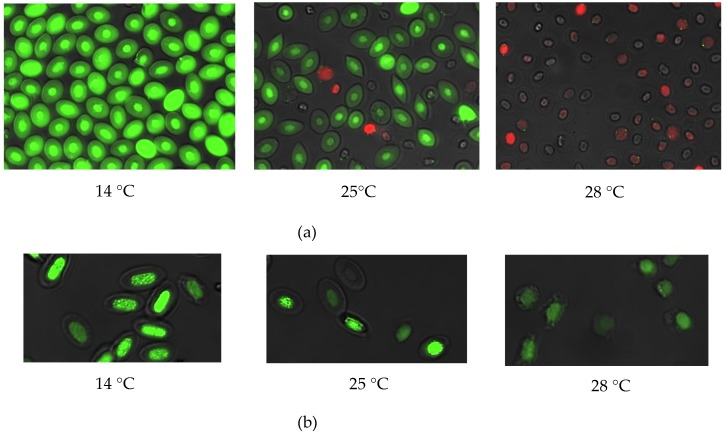
Evaluation of RBC and shRBC viability. The cell viability of RBCs and shRBCs obtained after incubation of RBCs at 14 °C, 25 °C and 28 °C, for 3 days was evaluated. (**a**) Representative fluorescence images of cells stained with the LIVE⁄DEAD^®^ Cell Vitality Assay Kit are shown. Live cells fluoresce bright green (substrate calcein AM), whereas dead cells with compromised membranes fluoresce red (EthD-1). (**b**) Representative fluorescence images of a 24 h EU-based viability assay in which EU (5-ethynyl uridine, 1 mM) is incorporated into nascent RNA and detected using Alexa Fluor^®^ 488 (green). Fluorescence images were taken with 60× magnification.

Cell viability of shRBCs was evaluated using the LIVE⁄DEAD^®^ Cell Vitality Assay Kit. Live cells fluoresce a bright green (substrate calcein AM), whereas dead cells with compromised membranes fluoresce red (EthD-1). Figure 3a shows representative images taken by fluorescence microscopy. RBCs showed bright green fluorescence without red nuclear staining. However, shRBCs showed some cells with red nuclear staining and other cells without red nuclear staining, but without calcein staining. EU (5-ethynyl uridine) (Click-iT^®^ RNA Alexa Fluor^®^ 488 imaging assay, green), an alkyne-modified nucleoside, was used as a viability marker since it is incorporated into newly synthesized RNA. An EU-based viability assay of RBCs and shRBCs showed EU-positive cells as green, with higher intensity results for RBCs (Figure 3b).

### 3.3. shRBCs Share Properties with Mammalian Primitive Pyrenocytes

**Figure 4:**
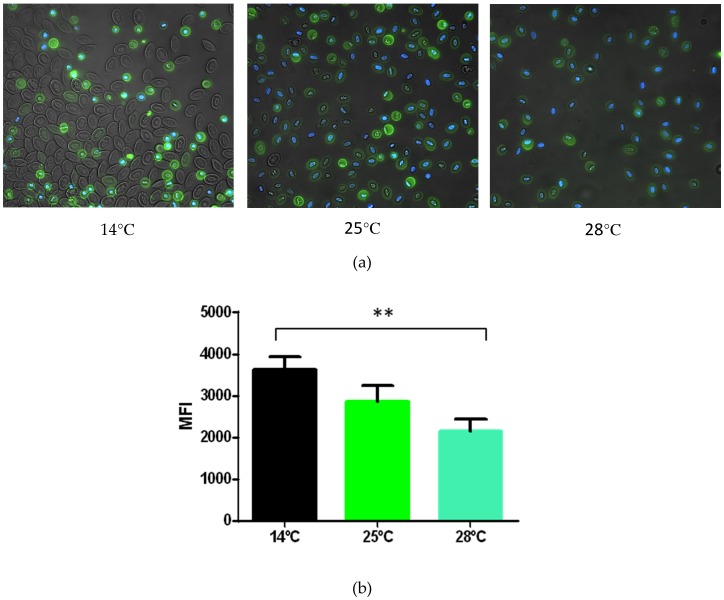
Phosphatidylserine expression on the membrane of RBCs and shRBCs. Ficoll-purified RBCs and shRBCs were obtained after incubation of RBCs at 14 °C, 25 °C and 28 °C, for 6 days. (**a**) Representative fluorescence microscopy images stained with annexin V and DAPI. (**b**) Mean fluorescence intensity (MFI) of annexin staining levels by flow cytometry. Data represent mean ± SD (n = 4). Kruskal–Wallis Test with Dunn’s Multiple Comparison post-hoc test was performed among all conditions. ** *p*-value < 0.01.

It became apparent that shRBCs exhibited morphology similarities consistent with mammalian primitive pyrenocytes (i.e., a membrane-encased nucleus surrounded by a thin rim of cytoplasm) [13]. One of the most characteristic features of mammalian pyrenocytes is that they expose PS on their surface as an “eat me” signal, resulting in engulfment and degradation by macrophages [16]. To explore this in vitro we studied PS exposure on the membrane of shRBCs obtained after incubation of RBCs at 14 °C, 25 °C and 28 °C. After 6 days, cells were stained with annexin V and representative images were taken by fluorescence microscopy (Figure 4a). Mean fluorescence intensity (MFI) of annexin V staining levels were measured by flow cytometry (Figure 4b) from the shRBC population, which was selected by FSC and SSC as indicated in Figure 7a. shRBCs were annexin V-positive, while RBCs were mainly annexin V-negative; however, annexin V staining in shRBCs was reduced as incubation temperature increased (Figure 4b). 

### 3.4. Transcriptome Evaluation of shRBCs

For a better understanding of how shRBCs differ from RBCs, we performed an exhaustive transcriptomic study to compare both cell stages. Ficoll-purified RBCs from two individuals were incubated at 14 °C and 28 °C for 3 days. After that, 14 °C RBCs and 28 °C shRBCs were single-cell sorted to obtain pure samples of each cell stage using BD FACSJazz™ FACS Sorter. Two samples of 20 to 30 cells each were sorted from RBCs and shRBCs and were used for RNA-seq. 

**Figure 5:**
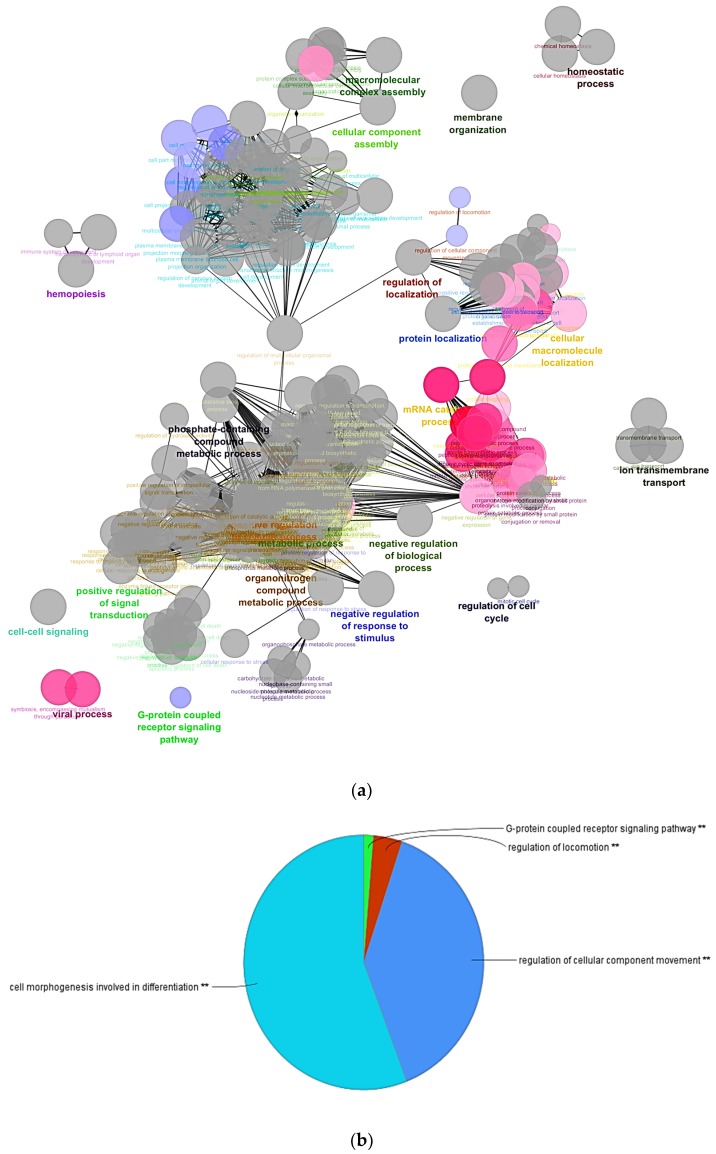

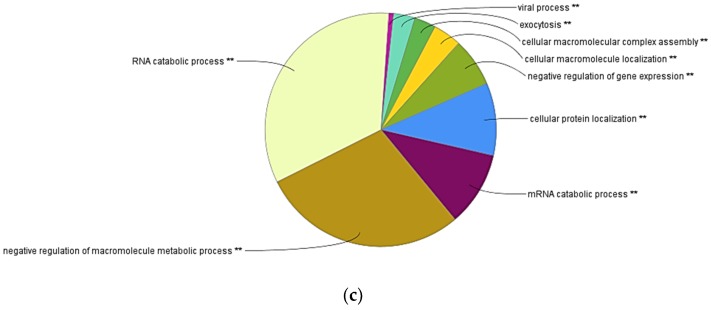
Functional pathway analysis of differentially expressed genes (DEGs) in shRBCs compared to RBCs. (**a**) Overrepresented GO terms were identified by Cytoscape ClueGo app, with Biological Process GO terms. Grey circles represent non-specific terms, red circles indicate overrepresented terms in RBCs, and blue circles indicate overrepresented terms in shRBCs. The biological process multilevel pie chart show (**b**) shRBCs and (**c**) RBCs overrepresented terms.

A total of 4946 out of the 139,195 genes were differentially expressed with FDR (false discovery rate) *q*-value <0.05 and *p*-value <0.05. Differential analysis revealed 1630 genes upregulated in RBCs and 1491 genes upregulated in shRBCs. Functional pathway enrichment analysis of differentially expressed genes (DEGs), was performed using Cytoscape ClueGO app with Biological Process GO terms. Common GO terms between RBCs and shRBCs were: (i) cellular protein metabolic process, (ii) organonitrogen compound metabolic process, (iii) positive regulation of cellular process, (iv) protein modification by small protein conjugation or removal, (v) protein localization, (vi) regulation of metabolic process, (vii) positive regulation of signal transduction, (viii) transport, (ix) homeostatic process, (x) hemopoiesis, and (xi) regulation of cell cycle (Figure 5a) (Supplementary Table S1). Overrepresented GO terms in shRBCs were: (i) cell morphogenesis involved in differentiation, (ii) regulation of cellular component movement, and (iii) regulation of locomotion and G-protein coupled receptor signaling pathway (Figure 5b) (Supplementary Table S1). Overrepresented GO terms in RBCs were: (i) RNA catabolic process, (ii) negative regulation of macromolecule metabolic process, (iii) mRNA catabolic process, (iv) cellular protein localization, (v) negative regulation of gene expression, vi) cellular macromolecule localization, (vii) cellular macromolecular complex assembly exocytosis, and viii) viral process (Figure 5c) (Supplementary Table S1).

Table 2 includes the most upregulated DEGs in shRBCs. Of special interest is the presence of genes encoding proteins involved in the modulation of DNA such as origin recognition complex subunit 2 (ORC2) and MORC family CW-type zinc finger 3 (MORC3). ORC2 is an essential component of the prereplication complex that binds to chromatin and initiates DNA replication [41]. MORC3 is a member of a highly conserved nuclear matrix protein superfamily and plays an important part in chromatin remodeling, DNA repair, epigenetic regulation, and cellular senescence [42,43]. Other genes of interest that were upregulated in shRBCs included SAM pointed domain containing Ets transcription factor (SPDEF), which is a transcription factor of the Ets family that is important in intestinal cell differentiation amongst other functions [44], and fucosyltransferase 9 (FUT9), which is involved in differentiation of mouse embryonic stem cells through increasing cell surface sialylation and fucosylation [45]. We also identified a highly upregulated group of genes involved in the modulation of ion channels such as guanine nucleotide-binding protein G(s) subunit alpha isoforms short (GNAS), disks large homolog 2 (DGL2), serum/glucocorticoid regulated kinase 2 (SGK2) and SH3 and multiple ankyrin repeat domains 3 (SHANK3). Specifically, SGK2 serves in diverse functions such as regulation of basic homeostatic functions like epithelial transport and regulation of excitability, cell volume, cell proliferation and apoptosis [46]. Strikingly, we found highly upregulated genes specific to other cell types such as T-cell ecto-ADP-ribosyltransferase 1 (Art2a), which is a protein expressed in murine T lymphocytes [47].

### 3.5. Proteomic Sequencing of shRBCs

**Figure 6:**
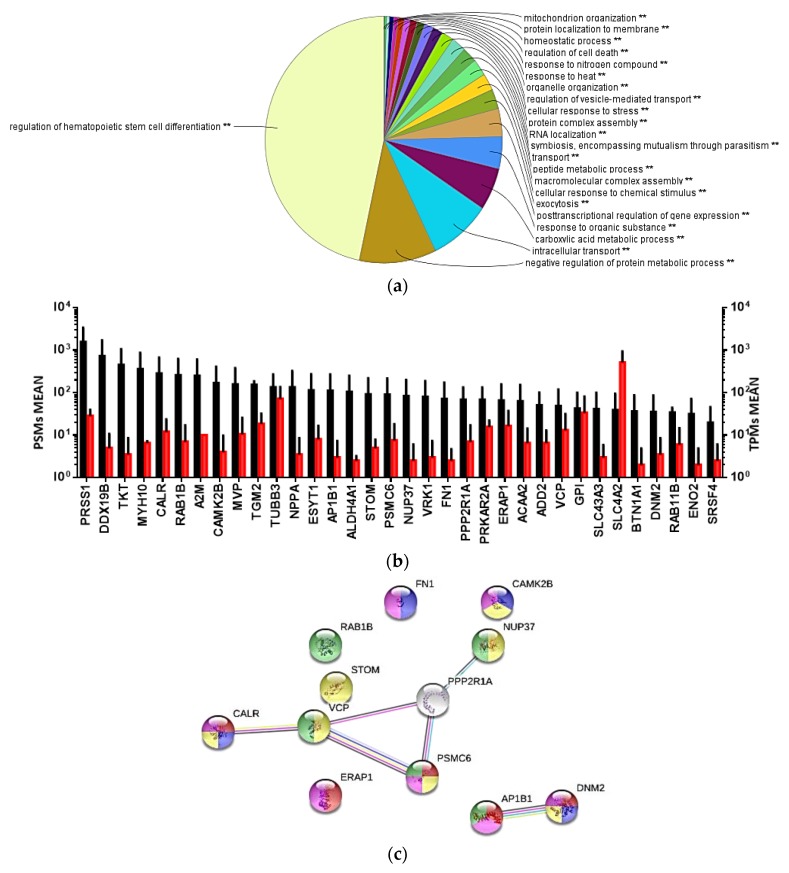
Combined transcriptome and proteome profiling in shRBCs. (**a**) Multilevel pie chart of overrepresented GO terms in the shRBC proteome profile, by means of the Cytoscape ClueGo app with Biological Process GO terms. (**b**) Common genes/proteins in the transcriptome and proteome analyses of shRBCs. Black bars represent TPM (transcript per million) and red bars represent PSMs (peptide spectrum match). Data represent mean ± SD (n = 5). (**c**) Protein-protein interaction network of genes/proteins related to the immune system. Node color highlights the proteins functionally annotated with STRING software: red, antigen processing and presentation of peptide antigen; blue, positive regulation of cell morphogenesis involve in differentiation; yellow, cellular response to stress; green, immune system process; and pink, viral process.

Proteome profiling of shRBCs identified 836 proteins, with 484 proteins having ≥2 peptide-to-spectrum matches (PSMs). Figure 6a shows a multilevel pie chart with overrepresented GO terms. The most represented GO terms were: (i) regulation of hematopoietic stem cell differentiation (45%), (ii) negative regulation of protein metabolic process, and (iii) intracellular transport and carboxylic acid metabolic process. Among the less represented GO terms included those in cellular responses to organic substance, chemical stimulus, stress, and heat (Supplementary Table S2).

Combined evaluation of proteome and transcriptome analyses is a crucial verification tool for the expression of key genes [48]. Combined transcriptome and proteome profiling of shRBCs resulted in 34 common proteins (Figure 6b). Functional annotation of these proteins (Supplementary Table S2) revealed 12 proteins involved in GO terms related to immune response, such as: (i) regulation of hematopoietic stem cell differentiation, (ii) viral process, and (iii) antigen processing and presentation of peptide antigen. The interactome network was built for these proteins to reveal protein-protein interactions and predict functional associations. The identified proteins highly interacted with each other with a PPI enrichment *p*-value < 0.05 and were involved in: (i) antigen processing and presentation of peptide antigen (AP1B1[adaptor related protein complex 1 beta 1 subunit], DNM2 [dynamin 2], ERAP1 [endoplasmic reticulum aminopeptidase 1], CALR [calreticulin], and PSMC6 [26S proteasome regulatory subunit 10B]; (ii) positive regulation of cell morphogenesis involves in differentiation (FN1 [fibronectin], CAMK2B [calcium/calmodulin-dependent protein kinase type II subunit beta], DNM2, and CALR); (iii) cellular response to stress (VCP [transitional endoplasmic reticulum ATPase], STOM [erythrocyte band 7 integral membrane protein], NUP37 [nucleoporin Nup37], CAMK2B, CALR, PSMC6, and DNM2); (iv) immune system process (ERAP1, DNM2, CAMK2B, FN1, CALR, and PSMC6); and (v) viral process (RAB1B [ras-related protein Rab-1B], VCP, AP1B1, NUP37, and PSMC6) (Figure 6c). 

### 3.6. Protein Cell Marker Screening in shRBCs

**Figure 7:**
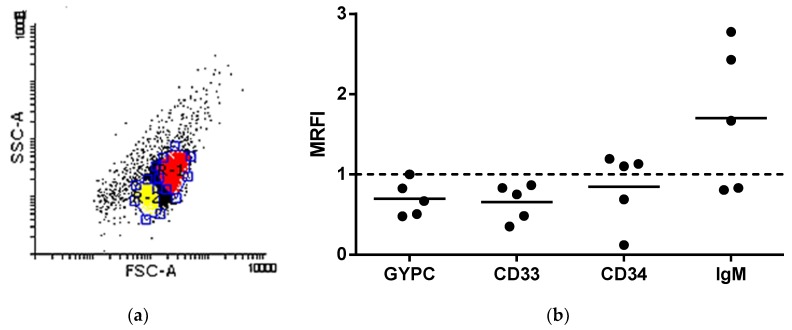
Protein cell markers for shRBCs. (**a**) RBCs incubated at 25 °C for 3 days were stained with different antibodies and analyzed by flow cytometry. The dot plot shows RBCs (red) and shRBCs (yellow). Flow cytometry results are represented as MRFI (mean relative fluorescence intensity = fluorescence in shRBC/RBC). (**b**) Protein cell markers: erythrocyte marker (glycophorin C [GYPC]), myeloid cell surface antigen (CD33), hematopoietic progenitor cell surface antigen (CD34), and B cell marker (immunoglobulin M [IgM]). Data are displayed as black circles showing the mean ± SD (n = 5). The Mann–Whitney Test was performed for statistical analysis between shRBCs and RBCs. RBCs are represented by the dashed line.

We observed that shRBCs are involved in the hematopoietic cell differentiation process. To further characterize shRBCs, a mixed population of RBCs and shRBCs, obtained after incubating RBCs at 25 °C for 3 days, was labeled using following antibodies for blood cell protein markers: (i) erythrocyte (glycophorin C [GYPC]); (ii) myeloid cell surface antigen (CD33); (iii) hematopoietic progenitor cell surface antigen (CD34); and (iv) B cell marker (immunoglobulin M [IgM]). After cell staining, samples were analyzed by flow cytometry. A dot plot depicted 2 populations: RBCs (red) with high FSC and shRBCs (yellow) with low FSC (Figure 7a). Results show a striking upregulation of IgM in shRBCs in comparison with RBCs. In addition, a slight and nonsignificant downregulation of GYPC, CD33, and CD34 was observed in shRBCs (Figure 7b). Thus, shRBCs appear to lose RBC markers such as GYPC. Surprisingly, new markers are apparent in shRBCs, such as IgM, a specific marker for B lymphocytes. 

### 3.7. shRBCs Immune Response to VHSV, a Viral Pathogen 

**Figure 8:**
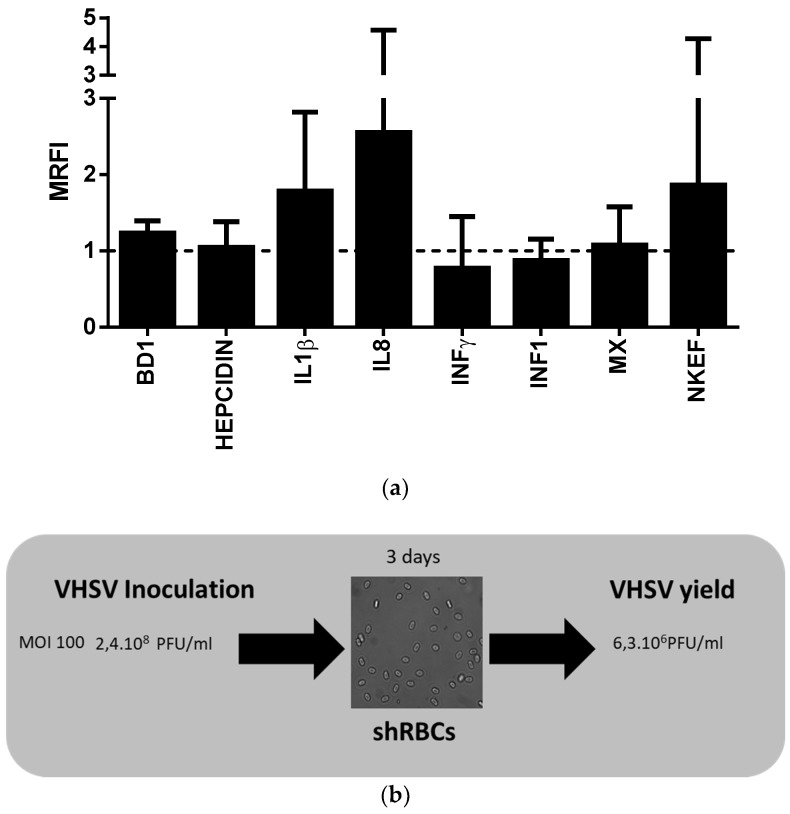
Antiviral immune response of shRBCs to VHSV exposure. (**a**) shRBCs, obtained after incubation of RBCs at 28 °C, for 3 days, were exposed to VHSV (MOI 100), at 14 °C, for 3 days. Protein levels of antimicrobial peptides (BD1 [beta defensin 1] and hepcidin), cytokines (IL8, IL1β and IFNγ), interferon-related proteins (IFN1 and Mx), and natural killer enhancing factor (NKEF) were evaluated in shRBCs and shRBCS exposed to VHSV by means of flow cytometry. Mean relative fluorescence intensity (MRFI) was calculated as the fluorescence intensity of VHSV-exposed shRBCs relative to unexposed control shRBCs. Data represent mean ± SD (n = 4). A Mann–Whitney Test was performed for statistical analysis between VHSV-exposed and control cells (dashed line). (**b**) Schematic representation of the VHSV infectivity in shRBCs, indicating virus inoculation titer and recovered virus yield after 3 days of exposure.

Because shRBCs express genes and proteins involved in immune system processes, we wanted to investigate whether shRBCs are able to mount an immune response to a viral attack, specifically VHSV. We analyzed protein expression of antimicrobial peptides (BD1 [β-defensin 1] and hepcidin), cytokines (IL1β [interleukin 1 β], IFNγ [interferon γ], and IL8 [interleukin 8]), proteins related to interferon response (IFN1 [interferon type 1] and Mx [myxovirus resistance protein]), and the natural killer enhancing factor (NKEF) using specific antibodies. In response to VHSV exposure, shRBCs slightly upregulated protein expression of IL1β, IL8, and NKEF in comparison with non-exposed shRBCs (Figure 8a). Moreover, results showed a decrease in viral yield titer in relation to inoculum titer (Figure 8b).

### 3.8. shRBCs CM Triggered TPS-2 Cytokine Signalling

**Figure 9:**
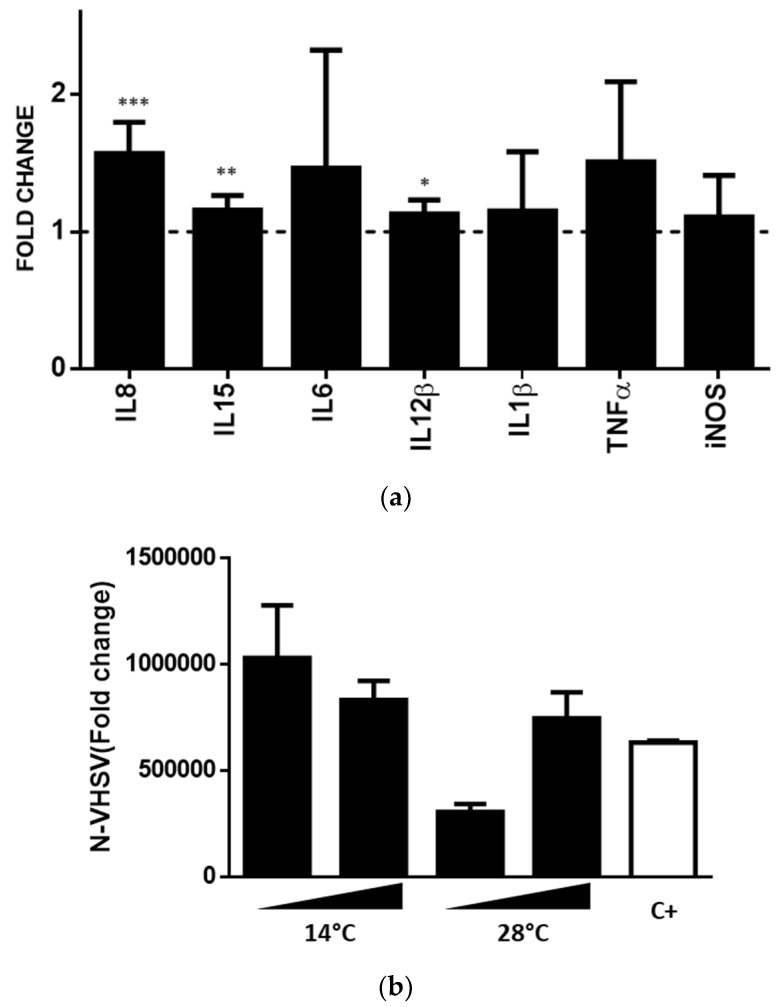
shRBCs CM triggered TPS-2 cytokine signaling. (**a**) Crosstalk between shRBCs CM (diluted 1/5 in RPMI 20% FBS) and TPS-2 cells was evaluated using RT-qPCR of IFNγ-activated genes (*il1b*, *il6*, *il12β*, *il15*, *tnfα*, *and inos*) and the chemokine *il8* gene. Gene expression was normalized against elongation factor 1 *α* (*ef1α*). Data represent mean ± SD (n = 4). A Mann–Whitney Test was performed for statistical analysis between VHSV-exposed and unexposed control cells (dashed line). *, **, and ***, represent *p*-values < 0.05, 0.01, and 0.001, respectively, compared to untreated control cells (dashed line). (**b**) TPS-2 cells were incubated with RBC and shRBC CM (diluted 1/5 and 1/125 in RPMI 20% FBS) for 24 h. Black triangles indicate increasing CM dilutions. Then, cells were infected with VHSV (MOI 1 × 10^−2^) at 14 °C for 24 h. VHSV infectivity was evaluated by RT-qPCR using specific primers for the N gene of VHSV (N-VHSV). Gene expression was normalized against *ef1α*. Data represent mean ± SD (n = 3). The Kruskal–Wallis Test with Dunn’s Multiple Comparison post-hoc test was performed among all conditions.

Given that shRBCs can produce immune proteins such as proinflammatory cytokines IL1β, IL8, and IFNγ (Figure 8a), we performed an experiment with shRBC CM obtained after RBC incubation at 14 °C and 28 °C for 3 days to investigate whether crosstalk between shRBCs and other immune cells occurs. A rainbow trout lympho-hematopoietic stromal cell line (trout pronephric stroma-2, TPS-2) was incubated with shRBC and RBC CM, for 1 day. IFNγ activated genes (*il1b*, *il6*, *il12β*, *il15*, *tnfα*, *and inos*) [49,50] and the gene encoding the chemokine *il8* were evaluated in TPS-2 cells using RT-qPCR. Results showed a significant upregulation of *il8*, *il15*, *il6*, *il12β*, and *tnfα* in TPS-2 cells incubated with CM of shRBCs (Figure 9a). Moreover, we assessed whether shRBC CM could confer protection against VHSV infection in TPS-2 cells. At 1/5 dilution, shRBC CM decreased VHSV infection in TPS-2 cells per N-VHSV gene RT-qPCR (Figure 9b).

### 3.9. shRBCs in Circulating Blood of Rainbow Trout

**Figure 10:**
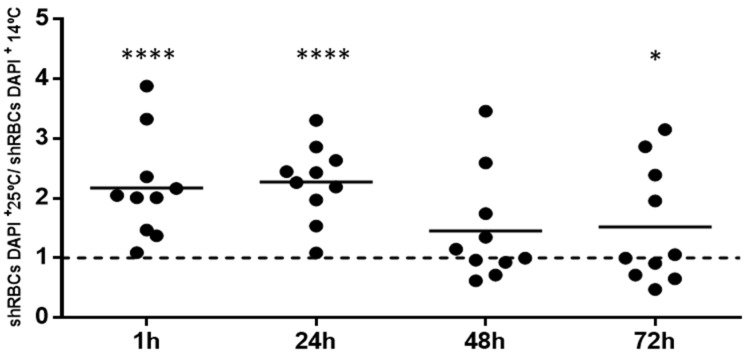
shRBCs in rainbow trout circulating blood. Fish were exposed to 25 °C for 1 h and then bled at 1, 24, 48, and 72 h post heat stress. DAPI-positive shRBCs were counted using the IN Cell Analyzer 6000 imaging system software. Data are displayed as black circles showing the mean ± SD (n = 10). The Kruskal–Wallis Test with Dunn’s Multiple Comparison post-hoc test was performed between all conditions. * and **** represent *p*-values < 0.05 and 0.0001, respectively, as compared to nonheat-stressed control cells (dashed line).

To investigate the possible presence of shRBCs in the circulating peripheral blood of rainbow trout, we performed an in vivo heat stress assay [22]. Fish were exposed to 25 °C for 1 h and then returned to normal temperature (14 °C). Blood was extracted at 1, 24, 48 and 72 h post heat stress. Blood cells were stained with DAPI and scanned by fluorescence microscopy. One observed property of shRBCs is the permeability of their membrane to DAPI as shown in Figure 4a; therefore, shRBC DAPI-positive cells were counted using IN Cell 6000 Analyzer software. DAPI-positive shRBC were increased in circulating peripheral blood at 1, 24, and 48 h post heat stress compared to unstressed fish (Figure 10). At 72 h post heat stress, the level of DAPI-positive cells was diminished to almost that of unstressed fish (Figure 10). However, DAPI-positive shRBCs were observed in circulating blood for at least 72 h post heat stress. 

## 4. Discussion

Rainbow trout Ficoll-purified RBCs cultured in vitro undergo morphological changes to a new cell stage or type that we have termed shRBCs. Morphological evolution of rainbow trout shRBCs in vitro varied among individuals, but their generation is evident when RBCs are exposed to some types of stress, such as temperature, mechanical or chemical stimulation. shRBCs are characterized by a rounded cell shape, a thin cell membrane, reduced cytoplasmic density, loss of hemoglobin, and a nucleus with decondensed chromatin. Light density cytoplasm is a characteristic feature of shRBCs, which differentiate them from reticulocytes whose cytoplasm is highly granular and have clusters of fibrous material that impart dark coloration to the cells in stained Hematoxylin/Eosin sections [51]. In addition, shRBCs expressed striking cell molecular markers, such as IgM, a specific marker for B lymphocytes. shRBCs share some characteristic morphological features of mammalian primitive pyrenocytes. However, in contraposition to pyrenocytes that rapidly disintegrate in cell culture [14], shRBCs were observed highly refractive in in vitro culture for more than a month. In rainbow trout, shRBCs were transiently observed in the blood circulation for 3 days following heat stress. PS exposure on the membrane of shRBCs was transient decreasing after several days of in vitro culture, suggesting that some PS-negative shRBCs could escape from phagocytes and therefore play a possible role in piscine immune response.

Differential transcriptomic analysis of shRBCs and RBCs revealed common GO terms within Biological Process categories. The most represented GO terms for both shRBCs and RBCs included cellular protein metabolic process, organonitrogen compound metabolic process, positive regulation of cellular process, protein modification by small protein conjugation or removal, protein localization, regulation of metabolic process, positive regulation of signal transduction, transport, homeostatic process, hemopoiesis and regulation of cell cycle. In addition, the majority of overrepresented shRBC GO terms were related to cell morphogenesis involve in differentiation (~60%). Moreover, among the 34 proteins common between the transcriptomic and proteomic analyses, 12 are involved in cell morphogenesis involved in differentiation, cellular response to stress, immune system process, and viral process. Similar results have been found in a transcriptomic analysis of red blood cells of rainbow trout exposed to heat stress [12], where the authors observed upregulation of genes related to the differentiation and development of blood cells, as well as genes involved in the stress and immune responses, at 24 h post stress. Moreover, in the transcriptome of Atlantic salmon RBCs, Dahle et al. [5] observed genes involved in DNA repair, responses to cellular stress, and erythroid differentiation, indicating that RBCs may retain the potential for differentiation and phenotype change.

Among the most upregulated genes in shRBCs, we found genes involved in DNA modulation such as MORC3 (a protein associated with chromatin remodeling) and ORC2 (a component of pre-replication complex proteins). In this regard, it has been reported that terminally differentiated *Xenopus* erythrocyte nuclei lack ORC1 and ORC2 proteins, rendering them unable to replicate and thus they remain proliferatively quiescent throughout their lifetime. However, quiescent nuclei from differentiated somatic cells can reacquire pluripotency (the capacity to replicate) and reinitiate a program of differentiation after transplantation into amphibian eggs [52]. Essential constituents of this transformation include the presence of components of pre-replication complex and permeabilization of the nuclear membrane, amongst others. shRBCs have a permeable nuclear membrane; this finding, together with high expression of ORC2 and MORC3, nucleus chromatin decondensation in shRBCs, and the expression of B cell type marker led us to assess the possibility that shRBCs may redifferentiate into another cell type. Moreover, the most represented GO term detected in the shRBC proteome sequencing was hematopoietic stem cells differentiation (45%), a result that reinforced previous findings. This evidence could explain the presence of different cell types when shRBCs are maintained long-term in vitro (data not shown). 

VHSV-exposed shRBCs exhibited an antiviral immune response characterized by an increase in IL8, IL1β, IFNɣ and NKEF proteins. In addition, shRBC CM induced communication with stromal TPS-2 cells and resulted in upregulation of IFNγ- activated genes (*il1b*, *il6*, *il12*, *il15*, *tnfα*, *and inos*) and *il8* gene in this cell line. shRBCs CM also induced protection against VHSV infection in TPS-2 cells. IFNγ is a highly pleiotropic pro-inflammatory and anti-viral cytokine exclusively produced in immune-related cells [49] and has been detected in murine nucleated erythroid cells [53]. It also has been shown that chicken erythrocytes stimulated with *C. albicans* release cytokine like-factors with IFNγ-like activity [54]. Moreover, chicken erythrocytes stimulated with poly I:C and CpG oligodeoxynucleotides (CpG ODN) showed an increase in IL8 transcripts [55]. Similarly, rainbow trout RBCs exposed to VHSV had increased IL8 protein levels [6]. It has also been reported that NKEF in human RBC cytosol mediates enhancement of NK cell activity [56]. All of this evidence appears to suggest that shRBCs share the roles of RBCs in immune responses. 

In conclusion, shRBCs may participate in roles related to (i) immune response mediation and homeostasis, as has been recently described for rainbow trout RBCs [6,8], and (ii) differentiation and development of blood cells. These ideas require further study, and we are continuing to investigate whether different shRBC populations contribute to these roles. Finally, a potential application of this study could be considering shRBCs as mediator cells of the immune response and therefore targets for novel DNA vaccines and/or new strategies against fish viral infections.

## Figures and Tables

**Table 1:** Primers and probes used in the study.

Gene	Forward primer (5′–3′)	Reverse primer (5′–3′)	Probe (5′–3′)	Reference or Accession Number
*ef1α*	ACCCTCCTCTTGGTCGTTTC	TGATGACACCAACAGCAACA	GCTGTGCGTGACATGAGGCA	[35]
*N-VHSV*	GACTCAACGGGACAGGAATGA	GGGCAATGCCCAAGTTGTT	TGGGTTGTTCACCCAGGCCGC	[36]
*il8*	AGAGACACTGAGATCATTGCCAC	CCCTCTTCATTTGTTGTTGGC	TCCTGGCCCTCCTGACCATTACTGAG	[37]
*ifnγ*	TCCAAGGACCAGCTGTTCAAC	TCATCAACACCCTCTGCTCACT	CCTGTTTTCCCCAAGGACACGTTTGAG	[38]
*tnfα*	AGCATGGAAGACCGTCAACGAT	ACCCTCTAAATGGATGGCTGCTT	AAAAGATACCCACCATACATTGAAGCAGATTGCC	[39]
*il1β*	GCCCCCAACCGCCTTA	CAGTGTTTGCGGCCATCTTA	ACCTTCACCATCCAGCGCCACAA	AJ278242.2
*il15*	TACTATCCACACCAGCGTCTGAAC	TTTCAGCAGCACCAGCAATG	TTCATAATATTGAGCTGCCTGAGTGCCACC	[6]
*il6*	ACTCCCCTCTGTCACACACC	GGCAGACAGGTCCTCCACTA	CCACTGTGCTGATAGGGCTGG	HF913655.1
*il12β*	TGACAGCCAGGAATCTTGCA	GAAAGCGAATGTGTCAGTTCAAA	ACCCAACGACCAGCCTCCAAGATG	AJ548829.1
*inos*	TCAGAACCTCCTCCACAA	GTGTACTCCTGAGAGTCCTTT	GCACCGACAGCGTCTA	AJ300555.1

**Table 2:** Top 20 most upregulated DEGs in shRBC RNA-seq analysis.

Gene Symbol	Gene Description	Log2 Fold Change
**MT-ND2**	NADH-ubiquinone oxidoreductase chain 2	17.462
**MICU1**	Calcium uptake protein 1, mitochondrial	16.460
**TFPI2**	Tissue factor pathway inhibitor 2	16.342
**ORC2**	Origin recognition complex subunit 2	15.899
**Art2a**	T-cell ecto-ADP-ribosyltransferase 1	15.871
**SLIT3**	Slit homolog 3 protein	15.443
**DLG2**	Disks large homolog 2	15.364
**PDZD2**	PDZ domain-containing protein 2	15.272
**SYT14**	Synaptotagmin-14	15.111
**PGM2**	Phosphoglucomutase-2	15.080
**GNAS**	Guanine nucleotide-binding protein G(s) subunit alpha isoforms short	14.969
**TRADD**	Tumor necrosis factor receptor type 1-associated DEATH domain protein	14.919
**TBX5**	T-box 5	14.700
**MUC2**	Mucin 2	14.648
**FUT9**	Fucosyltransferase 9	14.464
**SGK2**	Serum/glucocorticoid regulated kinase 2	14.451
**SHANK3**	SH3 and multiple ankyrin repeat domains 3	14.314
**SPDEF**	SAM pointed domain containing ets transcription factor	14.266
**MORC3**	MORC family CW-type zinc finger 3	14.213
**SNX14**	Sorting nexin 14	14.139

## Supplementary Materials

The following are available online at http://www.mdpi.com/2073-4409/7/4/31/s1, Figure S1: shRBC generation after RBC exposure to VHSV, laminarin, LPS, and Poly I:C. Ficoll-purified RBCs were incubated with VHSV at MOI 1, laminarin (0.5 µg/µL), LPS (1 µg/µL) and Poly I:C (30 µg/µL) for 3 days at 14 °C. Cells were stained with DAPI at a concentration of 0.3 pg/mL for 10 min. Data are displayed as black bars showing mean ± SD (n = 3) relative to untreated control cells (dashed line). Table S1: List of all overrepresented biological process GO terms in shRBC and RBC transcriptome profiling. Table S2: List of all overrepresented biological process GO terms in shRBC proteome profiling.
