# Supplementary material for: Shape-Shifted Red Blood Cells: A Novel Red Blood Cell Stage?
Source: Cells. 2018 Apr 19;7(4):31. doi: 10.3390/cells7040031 (PMC5946108; doi:10.3390/cells7040031)
Supplement: Supplementary file 1 [file cells-07-00031-s001.zip › cells-287412-supp-final_corrected.pdf]

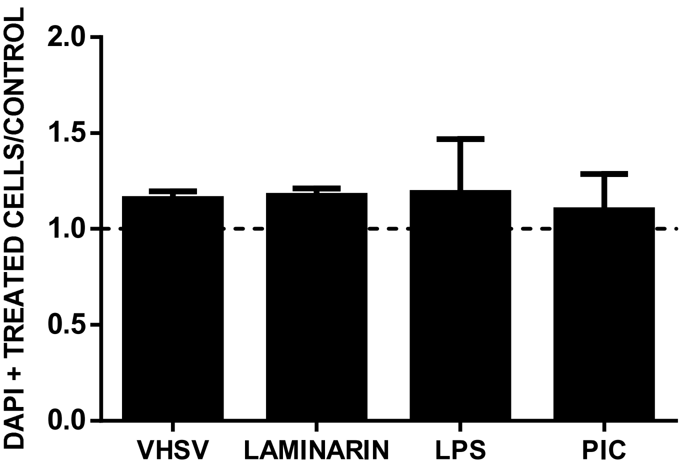

Figure S1. shRBC generation after RBC exposure to VHSV, laminarin, LPS, and Poly I:C. Ficoll-purified RBCs were incubated with VHSV at MOI 1, laminarin (0.5 µg/µL), LPS (1 µg/µL) and Poly I:C (30 µg/µL) for 3 days at 14 °C. Cells were stained with DAPI at a concentration of 0.3 pg/mL for 10 min. Data are displayed as black bars showing mean ± SD (n = 3) relative to untreated control cells (dashed line).
